# Ir76b is a Co-receptor for Amine Responses in *Drosophila* Olfactory Neurons

**DOI:** 10.3389/fncel.2021.759238

**Published:** 2021-11-17

**Authors:** Alina Vulpe, Karen Menuz

**Affiliations:** ^1^Department of Physiology and Neurobiology, University of Connecticut, Storrs, CT, United States; ^2^Connecticut Institute for Brain and Cognitive Sciences, University of Connecticut, Storrs, CT, United States

**Keywords:** olfactory, receptor, *Drosophila*, neuron, sensory, electrophysiology, ionotropic receptor (IR), amine

## Abstract

Two large families of olfactory receptors, the Odorant Receptors (ORs) and Ionotropic Receptors (IRs), mediate responses to most odors in the insect olfactory system. Individual odorant binding “tuning” OrX receptors are expressed by olfactory neurons in basiconic and trichoid sensilla and require the co-receptor Orco. The situation for IRs is more complex. Different tuning IrX receptors are expressed by olfactory neurons in coeloconic sensilla and rely on either the Ir25a or Ir8a co-receptors; some evidence suggests that Ir76b may also act as a co-receptor, but its function has not been systematically examined. Surprisingly, recent data indicate that nearly all coeloconic olfactory neurons co-express Ir25a, Ir8a, and Ir76b. Here, we demonstrate that Ir76b and Ir25a function together in all amine-sensing olfactory receptor neurons. In most neurons, loss of either co-receptor abolishes amine responses. In contrast, amine responses persist in the absence of Ir76b or Ir25a in ac1 sensilla but are lost in a double mutant. We show that responses mediated by acid-sensing neurons do not require Ir76b, despite their expression of this co-receptor. Our study also demonstrates that one population of coeloconic olfactory neurons exhibits Ir76b/Ir25a-dependent and Orco-dependent responses to distinct odorants. Together, our data establish the role of Ir76b as a *bona fide* co-receptor, which acts in partnership with Ir25a. Given that these co-receptors are among the most highly conserved olfactory receptors and are often co-expressed in chemosensory neurons, our data suggest Ir76b and Ir25a also work in tandem in other insects.

## Introduction

A vast number of different volatile molecules can be detected as odorants by both vertebrate and invertebrate animals. In insects, nearly all odorants are detected by receptors in one of two large gene families, the Odorant Receptors (ORs) and the Ionotropic Receptors (IRs; Vosshall and Stocker, 2007; Rytz et al., 2013). These receptors are found on the dendrites of olfactory receptor neurons (ORNs) housed in antennal olfactory sensilla. Large scale screens in *Drosophila* have shown that IR receptors generally detect polar compounds such as acids and amines, and they are expressed by ORNs in coeloconic sensilla (Benton et al., 2009; Silbering et al., 2011). Most ORNs express a single type of IrX “tuning” receptor that determines the odorants to which the neurons respond.

IRs are distantly related to well-characterized ionotropic glutamate receptors, such as AMPA and NMDA receptors (Benton et al., 2009). Like those receptors, IRs form ligand-gated non-selective cation channels, which are likely tetrameric complexes (Abuin et al., 2011). These complexes contain both IrX tuning subunits as well as highly conserved co-receptors that are required for the formation of functional receptors and trafficking of IrX to olfactory neuron dendrites (Abuin et al., 2011, 2019).

In *Drosophila*, there are four functional classes of coeloconic sensilla on the antennal surface. Each class contains one ORN that detects acidic odorants, and these responses require the co-receptor Ir8a (Yao et al., 2005; Abuin et al., 2011; Silbering et al., 2011). An acid-sensing ORN population in the sacculus also relies on this co receptor (Ai et al., 2013). The function of Ir8a is conserved in other insect species (Raji et al., 2019, Zhang J. et al., 2019).

Other ORNs in coeloconic sensilla respond to amines (Yao et al., 2005; Silbering et al., 2011), but here the co-receptor requirements are less clear. It was first reported that Ir25a is the required co-receptor for amine-sensing IRs based on the loss of amine responses mediated by ORNs in ac2 and ac4 sensilla in *Ir25a* mutants (Abuin et al., 2011). However, later studies found that amine responses mediated by Ir41a^+^ ORNs in ac2 sensilla require Ir76b, but not Ir25a, and that amine responses mediated by ac1 Ir92a^+^ ORNs are independent of IR co-receptors (Min et al., 2013; Hussain et al., 2016). Reconstitution experiments using ac4 amine receptor Ir76a demonstrated that both Ir25a and Ir76b are needed to form functional receptors (Abuin et al., 2011). However, coeloconic ORN amine sensitivity has not been systematically examined in Ir76b mutants, and the co-receptors for amine-sensing olfactory neurons remain unresolved.

Our understanding of co-receptor function has been further challenged with recent reports of co-receptor knock-in reporter lines that reveal a much broader expression of co-receptors than originally anticipated (Younger et al., 2020; Task et al., 2021). In *Drosophila*, it was found that most acid-sensing and amine-sensing coeloconic ORNs co-express Ir8a, Ir76b, and Ir25a (Task et al., 2021). Additionally, a handful of coeloconic ORNs strongly express all three IR co-receptors together with Orco, and many basiconic and trichoid ORNs express Orco and Ir25a (Benton et al., 2009; Task et al., 2021).

Here we examined the function of IR co-receptors in *Drosophila* olfaction using single-sensillum electrophysiology of co-receptor mutants. We discover that amine responses in all coeloconic sensilla rely on Ir25a and Ir76b, including responses mediated by ac3B ORNs. We further investigated the ac3B ORNs, which are known to express Orco and the tuning receptor Or35a, and showed that loss of co-receptors from one olfactory receptor family does not reduce responses mediated by the other olfactory receptor family within the same ORN.

## Materials and Methods

### Fly Stocks

*Drosophila* flies were raised on a standard cornmeal-molasses-agar food in a 25°C incubator with a 12:12 h light/dark cycle. Co-receptor mutant lines were obtained from the Bloomington Stock Center: #23130 *Orco^2^* (Larsson et al., 2004), #41744 *Ir8a^1^* (Abuin et al., 2011), #51309 *Ir76b^1^* (Zhang et al., 2013), and #41737 *Ir25a^2^* (Benton et al., 2009). Prior to experiments, each of these mutations was outcrossed for at least 10 generations into *wCS*, our standard genetic background that is a *Cantonized w^1118^* line (Koh et al., 2014). Such flies also served as the control flies (WT).

### Odorants

For most experiments, sensilla were recorded with a panel of 10 odorants and two solvents. Propionic acid (99%, ACROS Organics, CAS 79-09-4), 1,4-diaminobutane (99%, ACROS Organics, CAS 110-60-1), pyrrolidine (>99%, ACROS Organics, CAS 123-75-1), trimethylamine (~45%, Sigma–Aldrich, CAS 75-50-3), and ammonium hydroxide (28–30%, Fisher, CAS 1336-21-6) were diluted in water. Phenethylamine (99%, ACROS Organics, CAS 64-04-0), 1-hexanol (99%, ACROS Organics, CAS 111-27-3), 2-oxovaleric acid (>98%, Sigma–Aldrich, CAS 1821-02-9), phenylacetic acid (99%, Sigma–Aldrich, CAS 103-82-2), and phenylacetaldehyde (95%, Alfa Aesar, CAS 122-78-1) were diluted in paraffin oil. Other odorants were dimethylamine (~40%, Sigma–Aldrich, CAS 124-40-3) diluted in water for ac1 sensilla and amylamine (99%, Sigma–Aldrich, 110-58-7) diluted in paraffin oil for ac3 sensilla. Odorants were used at the following dilutions and concentrations: 1% propionic acid (134 mM), 1% 1,4-diaminobutane (112 mM), 1% pyrrolidine (120 mM), 1% trimethylamine (144 mM), 0.1% ammonium hydroxide (49 mM), 1% phenethylamine (73 mM), 0.001% 1-hexanol (85 μM), 1% 2-oxovaleric acid (77 mM), 1% phenylacetic acid (66 mM), 1% phenylacetaldehyde (74 mM), 1% dimethylamine (197 mM), and 0.32% amylamine (32 mM). Odorants were first diluted to 10%, then serially diluted to lower concentrations.

### Electrophysiology

Single-sensillum recordings (SSR) were performed on 2–5 day old female flies as previously described (Dobritsa et al., 2003; Benton and Dahanukar, 2011). Flies were inserted into a trimmed 200 μl pipette tip, with a portion of the head and both antennae exposed. A tapered glass electrode stabilized one antenna against a glass coverslip. The prep was visualized under a BX51WI microscope (Olympus) and kept under a 2 L/min humidified air stream applied through a glass tube. A borosilicate reference electrode and an aluminosilicate recording electrode were filled with recording solution (Kaissling, 1995). The reference electrode was inserted into an eye with a manual micromanipulator while the recording electrode was inserted into an individual sensillum with a MP-225 motorized micromanipulator (Sutter Instruments). An EXT-02F amplifier (NPI) with a custom 10x gain headstage was used for recording extracellular action potentials AC filtered at 300–1,700 Hz. A PowerLab 4/35 digitizer and LabChart Pro v8 software (ADInstruments) were used to acquire data at 10 kHz.

Odorant cartridges were made by pipetting 50 μl of odorant onto a 13 mm antibiotic assay disc (Whatman) inserted into a Pasteur pipette and closing the end of the pipette with a 1,000 μl pipette tip. Odorants were allowed to equilibrate for at least 20 min prior to experiments. Odorants were applied by opening a Lee valve (02-21-08i) that allowed a 0.5 L/min air stream to travel through the cartridge and into the main air stream through a hole in the glass tube. Odorant delivery was controlled by a ValveBank 4 controller (Automate Scientific), which was driven by LabChart. Each LabChart trace was 10 s long, with a 1 s baseline, followed by 0.5 s odorant application and 8.5 s recovery period. In figures, the bar above the traces indicates the time during which the valve was open. There is a brief odorant-dependent travel delay for the odorants to reach the sensillum, and thus the neuron firing response starts slightly later. Following the 10 s recording, an additional recovery period was provided before the application of another odorant to the same sensillum. Each sensillum was tested with the solvent control as well as multiple odorants. The spontaneous firing rate did not substantially change in response to solvent, with the exception of a small response to water in ac3 sensilla (Supplementary Figure 1). Each odorant was applied once per sensillum. Up to four sensilla were recorded per fly. Individual cartridges were allowed to recover for at least 10 min between odorant applications and were used up to four times before being discarded.

Responses were detected and analyzed using the LabChart Spike Histogram module. For reference, expanded traces are shown in Supplementary Figures 2–5. Action potentials were counted over a 0.5 s response window, which was 100 ms after the stimulus onset due to the line delay. The action potentials of individual ORNs could not be sorted due to the similarity of their amplitudes (Silbering et al., 2011), and therefore all spikes during the response period were counted in each sensillum. The odorant response was calculated as the response to the odorant minus the response to the solvent, either water or paraffin oil depending on the odorant. Sensillar classes were determined by their stereotypical location on the surface of the antenna and their responses to diagnostic odorants.

### Statistical Analysis

Data were analyzed using GraphPad Prism 9. Box plots depict the median and interquartile range, and the whiskers span the minimum to maximum data points. All individual data points are overlaid on the box plots. Mann-Whitney tests were used to compare two genotypes. For comparisons of four genotypes, a Kruskal-Wallis test was used and the false discovery rate was controlled with the two-stage linear step up method of Benjamini, Krieger, and Yekutieli. Sample sizes are found in the figure captions. Genotypes that are significantly different (*p* < 0.05) are indicated with different letters above the box plots.

## Results

### Amine Responses in ac2 and ac4 Sensilla Require Both Ir25a and Ir76b

We first examined the co-receptor dependence of responses to amines in ac2 and ac4 sensilla, which are mediated by ORNs expressing Ir41a (ac2), Ir76a (ac4), and Ir75d (ac2 and ac4; Silbering et al., 2011). We performed single-sensillum recordings (SSR) to compare odor-induced spiking responses in sensilla from co-receptor mutants with those from WT flies, utilizing odorants known to primarily activate specific neurons. The *Ir76b^1^*, *Ir25a^2^*, and *Ir8a^1^* mutations have been previously characterized (Benton et al., 2009; Abuin et al., 2011; Zhang et al., 2013). In *Ir25a^2^* and *Ir8a^1^*, the entire coding sequence was replaced with the *w* gene, whereas in *Ir76b^1^* approximately 50% of the coding region was deleted including two of three transmembrane domains and the pore re-entry loop (Benton et al., 2009; Abuin et al., 2011; Zhang et al., 2013).

**Figure 1:**
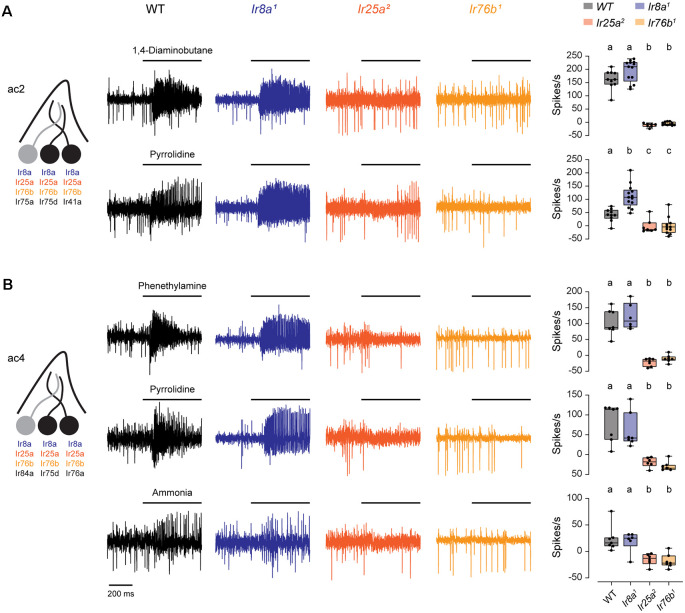
Both Ir25a and Ir76b are required co-receptors for amine-sensing receptors in ac2 and ac4 sensilla. **(A)** Left, diagram of an ac2 sensillum, showing receptor expression in each ORN. ORNs that respond to the tested amines are in black. Middle, representative traces of responses to 1,4-diaminobutane and pyrrolidine in ac2 sensilla in WT, *Ir8a^1^*, *Ir25a^2^*, and *Ir76b^1^* fly lines. Right, box plots overlaid with individual data points (*n* = 10 WT, 13 *Ir8a^1^*, 7 *Ir25a^2^*, and 11 *Ir76b^1^* sensilla). Genotypes that are significantly different are indicated with different letters. **(B)** Similar to **(A)**, but for ac4 sensilla tested with phenethylamine, pyrrolidine, and ammonia (phenethylamine: *n* = 7 WT, 6 *Ir8a^1^*, 7 *Ir25a^2^*, and 7 *Ir76b^1^* sensilla; pyrrolidine: *n* = 7 WT, 7 *Ir8a^1^*, 6 *Ir25a^2^*, and 7 *Ir76b^1^* sensilla; ammonia: *n* = 7 WT, 6 *Ir8a^1^*, 6 *Ir25a^2^*, and 7 *Ir76b^1^* sensilla). ORN, olfactory receptor neuron.

We found that responses to both pyrrolidine and 1,4-diaminobutane, mediated by Ir75d and Ir41a, respectively, were eliminated in both Ir25a and Ir76b mutants in ac2 sensilla (Figure 1A). Likewise, excitatory responses in ac4 sensilla to phenethylamine and ammonia, mediated by Ir76a, and to pyrrolidine, mediated by Ir75d, were lost in both Ir25a and Ir76b mutants (Figure 1B). Responses were not reduced in Ir8a mutants, although pyrrolidine responses were somewhat larger than the control line in ac2 sensilla. In each case, responses to solvents were minimal (Supplementary Figure 1), and odorant responses were calculated as the response to the odorant minus the response to the odorant solvent (see “Materials and Methods” section). Together, this suggests that only Ir25a and Ir76b are necessary for the proper functioning of these amine receptors, despite co-expression of Ir8a in these neurons.

### Either Ir25a or Ir76b is Sufficient to Maintain Amine Responses Mediated by Ir92a

**Figure 2:**
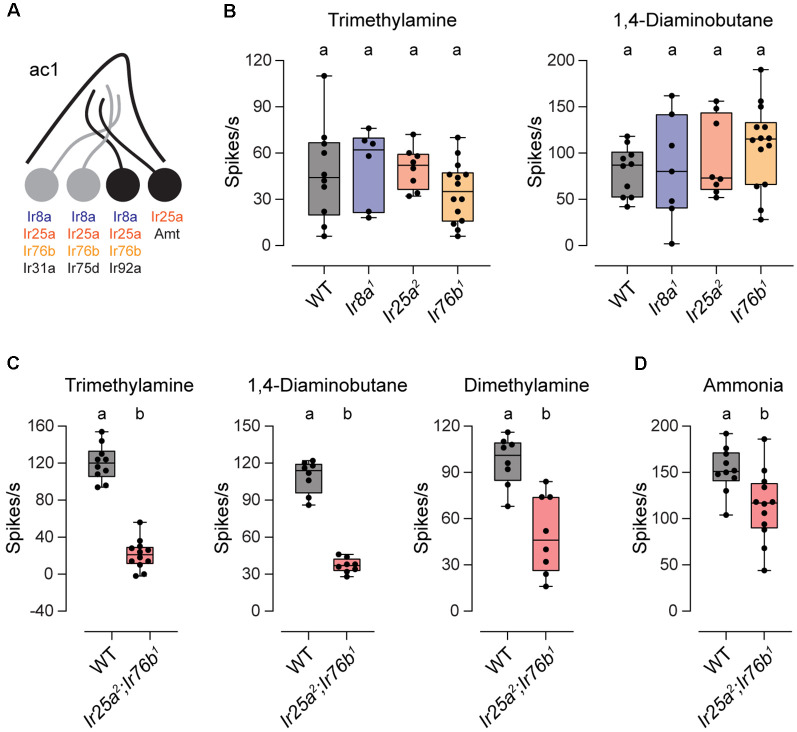
Ir25a and Ir76b play a redundant role in ac1 sensilla. **(A)** Diagram of an ac1 sensillum, showing receptor expression in each ORN. Those neurons that respond to the tested odorants are in black. **(B)** Box plots showing the distribution of responses to trimethylamine and 1,4-diaminobutane in ac1 sensilla in WT, *Ir8a^1^*, *Ir25a^2^*, and *Ir76b^1^* flies (trimethylamine: *n* = 10 WT, 6 *Ir8a^1^*, 8 *Ir25a^2^*, and 14 *Ir76b^1^* sensilla; 1,4-diaminobutane: *n* = 10 WT, 7 *Ir8a^1^*, 8 *Ir25a^2^*, and 14 *Ir76b^1^* sensilla). **(C)** Box plots showing the distribution of responses to trimethylamine, 1,4-diaminobutane, and dimethylamine in ac1 sensilla in WT and *Ir25a^2^*;*Ir76b^1^* flies (trimethylamine: *n* = 10 WT and 12 *Ir25a^2^*;*Ir76b^1^* sensilla; 1,4-diaminobutane: *n* = 8 sensilla each; dimethylamine: *n* = 8 sensilla each). **(D)** Box plot showing the distribution of responses to ammonia in WT and *Ir25a^2^*;*Ir76b^1^* flies (*n* = 10 WT and 12 *Ir25a^2^*;*Ir76b^1^* sensilla). Genotypes that are significantly different are indicated with different letters.

We also examined the co-receptor dependence of amine responses in ac1 sensilla. These sensilla contain four neurons, three of which respond to amines or to the related compound ammonia (Figure 2A). One neuron expresses Ir75d, similar to ac2 and ac4 sensilla, whereas Ir92a ORNs respond to a large variety of amines (Silbering et al., 2011; Min et al., 2013; Vulpe et al., 2021). Intriguingly, Ir92a signaling was shown to be independent of Ir25a, Ir76b, and Ir8a, despite the expression of all three co-receptors in these neurons (Benton et al., 2009; Min et al., 2013; Task et al., 2021). We verified these findings by measuring ac1 responses to trimethylamine and 1, 4-diaminobutane, two amines that activate Ir92a ORNs (Min et al., 2013; Vulpe et al., 2021). As reported, amine responses were retained in each IR co-receptor mutant (Figure 2B).

We wondered whether Ir25a and Ir76b play a redundant role in Ir92a neurons. To test this possibility, we generated a double-mutant *Ir25a^2^*;*Ir76b^1^* fly line. Responses to trimethylamine and 1,4-diaminobutane were reduced 82% and 66%, respectively, in the absence of these two co-receptors (Figure 2C). Likewise, responses to dimethylamine, a third odorant that activates Ir92a “ORNs (Silbering et al., 2011; Min et al., 2013; Vulpe et al., 2021), were reduced by 49% in the absence of both Ir25a and Ir76b (Figure 2C). Together our data indicate that all amine-sensing IR receptors, including Ir92a, rely on Ir25a and Ir76b, with most requiring both co-receptors, and Ir92a able to function with either co-receptor alone.

Two neurons in ac1 sensilla respond to ammonia, those that express Ir92a and those that express Amt, an ammonium transporter that serves as an olfactory receptor (Vulpe et al., 2021). Unlike ac1 responses to amines, ammonia responses were only slightly reduced (~23%) in *Ir25a^2^*;*Ir76b^1^* flies (Figure 2D). The reduced portion likely represents the response mediated by Ir92a ORNs because Amt ORNs function independently of IR receptors (Vulpe et al., 2021).

### OR and IR Receptors Are Utilized by ac3B ORNs to Respond to Distinct Odorants

**Figure 3:**
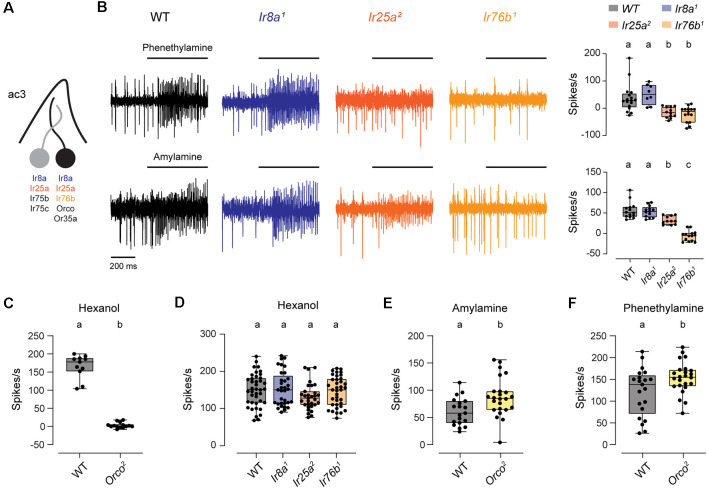
The ac3B ORNs utilize IR and OR receptors. **(A)** Diagram of an ac3 sensillum, showing receptor expression in each ORN. The ac3B ORN is in black. **(B)** Representative traces of responses to phenethylamine and amylamine in ac3 sensilla from WT, *Ir8a^1^*, *Ir25a^2^*, and *Ir76b^1^* flies. Right, box plots overlaid with individual data points (phenethylamine: *n* = 17 WT, 8 *Ir8a^1^*, 12 *Ir25a^2^*, and 15 *Ir76b^1^* sensilla; amylamine: *n* = 14 WT, 13 *Ir8a^1^*, 11 *Ir25a^2^*, and 14 *Ir76b^1^* sensilla). **(C)** Box plot of ac3 responses to hexanol in WT and *Orco^2^* flies (*n* = 11 WT and 14 *Orco^2^* sensilla). **(D)** Box plot of ac3 responses to hexanol in WT flies and IR co-receptor mutants (*n* = 40 WT, 33 *Ir8a^1^*, 31 *Ir25a^2^*, and 37 *Ir76b^1^* sensilla). **(E,F)** Box plot of responses to amylamine **(E)** and phenethylamine **(F)** in WT flies and *Orco^2^* mutants (amylamine: *n* = 19 WT and 24 *Orco^2^* sensilla; phenethylamine: *n* = 21 WT and 24 *Orco^2^* sensilla). Genotypes that are significantly different are indicated with different letters. OR, Odorant Receptor; IR, Ionotropic Receptor.

The ac3B ORN is unique in that it is the only coeloconic ORN known to express an OR family tuning receptor, Or35a (Couto et al., 2005; Yao et al., 2005). Or35a mediates responses to nearly all odorants detected by this neuron, and it does so together with the co-receptor Orco (Yao et al., 2005; Silbering et al., 2011). Interestingly, ac3B also expresses Ir76b, Ir8a, and Ir25a, despite the lack of a known IrX tuning receptor expressed by this coeloconic ORN (Figure 3A; Benton et al., 2009; Silbering et al., 2011; Task et al., 2021). Examining a previous dataset (Silbering et al., 2011), we identified two amines that lead to spiking in ac3 sensilla in the absence of Orco. We found that responses to phenethylamine were lost in *Ir25a^2^* and *Ir76b^1^* mutants, and responses to amylamine were lost in *Ir76b^1^* mutants and partially reduced in *Ir25a^2^* mutants (Figure 3B). Based on the characteristically small amplitude spikes, these responses were mediated by the ac3B ORN. Thus, Ir76b and Ir25a likely function as amine co-receptors for an unidentified IrX tuning receptor in these ORNs.

The ac3B ORN is one of the first olfactory neurons clearly shown to utilize receptors from both the IR and OR family. We, therefore, examined the impact of co-receptors from one olfactory receptor family on signaling mediated by the other family. Responses to hexanol are known to be mediated by Or35a/Orco (Yao et al., 2005; Silbering et al., 2011), and we verified this in *Orco^2^* mutants (Figure 3C). Responses to hexanol were unaffected by the loss of IR co-receptors, with no change compared to WT in *Ir8a^1^*, *Ir25a^2^*, and *Ir76b^1^* mutants (Figure 3D). We then examined responses to the IR-dependent odorants, amylamine and phenethylamine, in *Orco^2^* mutants. Responses to amylamine and phenethylamine were increased to varying degrees in *Orco^2^* mutants (amylamine: 47% higher than controls; phenethylamine: 12% higher than controls; Figures 3E,F), indicating that Orco is not required for the responses for IR-dependent odorants. The increased responses in the absence of Orco may suggest that there are interactions between the two signaling pathways; alternatively, IR-dependent odorants may directly inhibit the Or35a/Orco receptor.

### Ir8a is the Only Co-receptor Required for Responses in Acid-Sensing Neurons

Responses to acidic odorants are mediated by olfactory neurons expressing Ir31a (ac1), Ir75a (ac2), Ir75b and Ir75c (ac3), and Ir84a (ac4; Silbering et al., 2011; Prieto-Godino et al., 2017). The function of these receptors depends on Ir8a, but not Ir25a (Abuin et al., 2011). However, recent data indicate that Ir76b is co-expressed in most of these ORNs (Task et al., 2021). Given that the role of Ir76b in these neurons has not been tested previously, we examined the Ir76b-dependence of their odorant responses.

**Figure 4:**
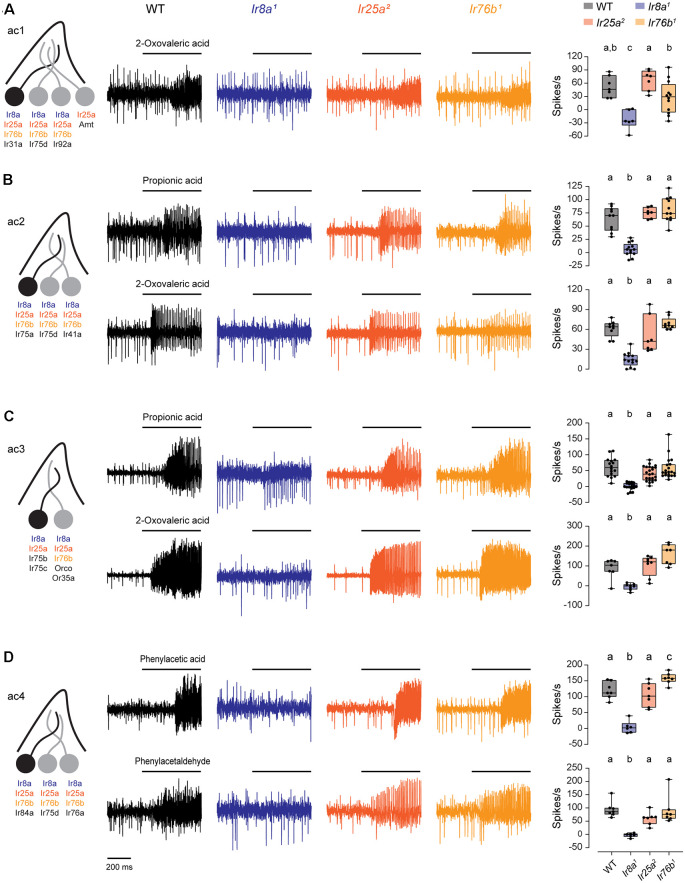
Ir8a is the sole co-receptor required for the function of acid-sensing receptors. **(A)** Left, diagram of an ac1 sensillum and receptor expression. The acid-sensing ORN is in black. Middle, representative traces of responses to 2-oxovaleric acid in ac1 sensilla in WT, *Ir8a^1^*, *Ir25a^2^*, and *Ir76b^1^* flies. Right, box plots overlaid with individual data points (*n* = 7 WT, 6 *Ir8a^1^*, 7 *Ir25a^2^*, and 12 *Ir76b^1^* sensilla). **(B)** Similar to **(A)**, but for ac2 sensilla tested with propionic acid and 2-oxovaleric acid (propionic acid: *n* = 10 WT, 13 *Ir8a^1^*, 7 *Ir25a^2^*, and 11 *Ir76b^1^* sensilla; 2-oxovaleric acid: *n* = 9 WT, 13 *Ir8a^1^*, 7 *Ir25a^2^*, and 9 *Ir76b^1^* sensilla). **(C)** Similar to **(A)**, but for ac3 sensilla tested with propionic acid and 2-oxovaleric acid (propionic acid: *n* = 16 WT, 18 *Ir8a^1^*, 24 *Ir25a^2^*, and 20 *Ir76b^1^* sensilla; 2-oxovaleric acid: *n* = 7 WT, 8 *Ir8a^1^*, 8 *Ir25a^2^*, and 7 *Ir76b^1^* sensilla). **(D)** Similar to **(A)**, but for ac4 sensilla tested with phenylacetic acid and phenylacetaldehyde (*n* = 7 WT, 6 *Ir8a^1^*, 7 *Ir25a^2^*, and 7 *Ir76b^1^* sensilla for each odorant). Genotypes that are significantly different are indicated with different letters.

We measured SSR responses to odorants known to be detected by acid-sensing ORNs in IR co-receptor mutants (Grosjean et al., 2011; Silbering et al., 2011). In ac1, we tested 2-oxovaleric acid, mediated by Ir31a (Figure 4A). In ac2, we tested propionic acid and 2-oxovaleric acid, mediated by Ir75a (Figure 4B). In ac3, we tested responses mediated by Ir75b and Ir75c to propionic acid and 2-oxovaleric acid (Figure 4C). In ac4, we tested phenylacetic acid and phenylacetaldehyde, which are detected by Ir84a (Figure 4D). Responses to each of these odorants were entirely eliminated in *Ir8a^1^* mutants, consistent with previous findings (Abuin et al., 2011). In contrast, responses did not require Ir76b or Ir25a (Figure 4, Supplementary Figure 6). Thus, Ir76b does not contribute to responses mediated by acid-sensing receptors.

## Discussion

This study clarifies the roles of IR co-receptors in olfactory neurons in *Drosophila*. We demonstrate that Ir76b functions together with Ir25a as a co-receptor for each of the IrX tuning receptors that detect amines, and that it does not contribute to acid-sensing receptor function. Further, we demonstrate that ac3B ORNs have Ir76b-dependent amine responses, in addition to their known Or35a/Orco-dependent odorant responses.

Our data indicate that each of the four classes of coeloconic sensilla has one ORN that responds to acids and one to three ORNs that responds to amines. Despite the co-expression of all three IR co-receptors in most of these neurons, the IR co-receptor dependence is largely segregated. The acid-sensing receptors only rely on the co-receptor Ir8a, whereas amine-sensing receptors rely on Ir25a and Ir76b. Interestingly, while responses to amines require both Ir25a and Ir76b in ac2, ac3, and ac4 sensilla, the co-receptor dependence of ac1 amine-sensing Ir92a ORNs differs. In these neurons, responses are only lost when both Ir25a and Ir76b co-receptors are absent, suggesting that Ir92a can form functional receptors with either Ir25a or Ir76b. This is intriguing because only Ir25a and Ir8a contain the amino-terminal domain involved in the assembly of IR subunits into heteromeric complexes (Croset et al., 2010; Abuin et al., 2019).

Our data showing that ac3B ORNs respond to amines in an IR co-receptor dependent fashion implies that there is a currently unidentified IrX tuning receptor in these neurons. Examination of our previous antennal RNASeq datasets reveals trace expression of a handful of candidate receptors that are consistently expressed in wild-type antennae, but lost in *atonal* mutants that lack coeloconic sensilla (Menuz et al., 2014; Mohapatra and Menuz, 2019). These candidates include *Ir60a*, *Ir94b*, *Ir100a*, *Ir56a*, and *Ir10a*. However, transgenic reporter lines for these receptors do not drive expression in antennal neurons, with the possible exception of *Ir56a* (Sanchez-Alcaniz et al., 2018).

Odorant-induced activation of OR and IR receptors within ac3B ORNs is likely to be functionally independent. We found that hexanol responses mediated by Orco/Or35a were unaffected by the absence of IR co-receptors, and amine responses were not reduced in an *Orco^2^* mutant. However, some interactions may occur given that amine responses were somewhat enhanced in *Orco^2^* flies. Multiple mechanisms could underlie this interaction. One possibility is that in addition to activating the IR tuning receptor, these amines also directly inhibit spontaneously open Or35a/Orco receptors. In the absence of Orco, the net effect of amines on the neuron’s spiking rate would be larger. Alternatively, inhibitory electrical interactions may occur between the two receptors in the same neuron, similar to the lateral inhibition that occurs between neurons in the same sensillum (Su et al., 2012; Zhang Y. et al., 2019).

An overall picture is emerging of Ir25a and Ir76b often working together as co-receptors in chemosensory neurons. In addition to our findings in the olfactory system, studies measuring activity in the primary gustatory neurons revealed that both co-receptors are necessary for responses to fatty acids, sour and carbonation (Ahn et al., 2017; Chen and Amrein, 2017; Sanchez-Alcaniz et al., 2018). However, other studies examining gustatory responses to amino acids, salt, and polyamines using behavioral assays found that these rely on Ir76b, but not Ir25a (Zhang et al., 2013; Croset et al., 2016; Hussain et al., 2016; Ganguly et al., 2017). Given that olfactory behaviors driven by polyamines show a similar selective dependence on Ir76b (Hussain et al., 2016), despite additional Ir25a-dependence of spiking responses in the polyamine sensitive Ir41a ORNs, it may be informative to directly test the role of Ir25a in the spiking responses of primary gustatory neurons to amino acids, salt, and polyamines. Our data suggest that a role for Ir25a and Ir76b cannot be ruled out by testing single mutants, given that they play a functionally redundant role in at least one class of neurons.

## Data Availability Statement

The original contributions presented in the study are included in the article and Supplementary Material. Further inquiries can be directed to the corresponding author.

## Author Contributions

KM and AV contributed to experimental design, analysis, and interpretation of results. AV performed electrophysiological recordings. KM wrote the manuscript with input from AV. All authors contributed to the article and approved the submitted version.

## Conflict of Interest

The authors declare that the research was conducted in the absence of any commercial or financial relationships that could be construed as a potential conflict of interest.

## Publisher’s Note

All claims expressed in this article are solely those of the authors and do not necessarily represent those of their affiliated organizations, or those of the publisher, the editors and the reviewers. Any product that may be evaluated in this article, or claim that may be made by its manufacturer, is not guaranteed or endorsed by the publisher.
