# Supplementary material for: Ir76b is a Co-receptor for Amine Responses in Drosophila Olfactory Neurons
Source: Front Cell Neurosci. 2021 Nov 17;15:759238. doi: 10.3389/fncel.2021.759238 (PMC8635857; doi:10.3389/fncel.2021.759238)
Supplement: Supplementary file 1 [file Data_Sheet_1.PDF]

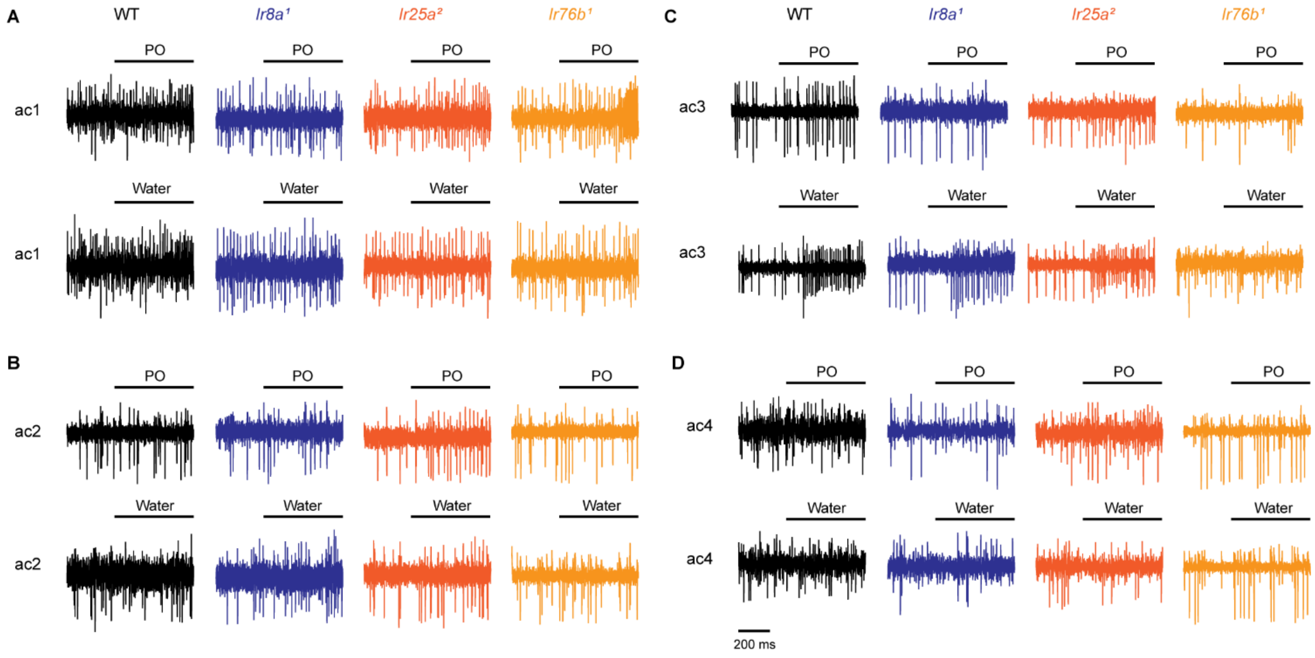

Supplementary Figure S1. Responses to solvents in ac1-ac4 sensilla. **(A)** Spiking responses to paraffin oil (PO) and water in ac1 sensilla in WT, *Ir8a^{1}*, *Ir25a^{2}*, and *Ir76b^{1}* flies. The PO traces are those from sensilla used for representative traces for 2-oxovaleric acid in Figure 4. The water responses were taken from sensilla tested with ammonia. **(B)** Spiking responses to PO and water in ac2 sensilla in WT, *Ir8a^{1}*, *Ir25a^{2}*, and *Ir76b^{1}* flies. The PO traces are those from sensilla used for representative traces for 2-oxovaleric acid in Figure 4, and the water traces are those from sensilla used for representative traces for pyrrolidine in Figure 1. **(C)** Spiking responses to PO and water in ac3 sensilla in WT, *Ir8a^{1}*, *Ir25a^{2}*, and *Ir76b^{1}* flies. The PO traces are those from sensilla used for representative traces for phenethylamine in Figure 3, and the water traces are those from sensilla used for representative traces for propionic acid in Figure 4. **(D)** Spiking responses to PO and water in ac4 sensilla in WT, *Ir8a^{1}*, *Ir25a^{2}*, and *Ir76b^{1}* flies. The PO traces are those from sensilla used for representative traces for phenethylamine responses, and the water traces are those from sensilla used as representative traces for ammonia, both in Figure 1.

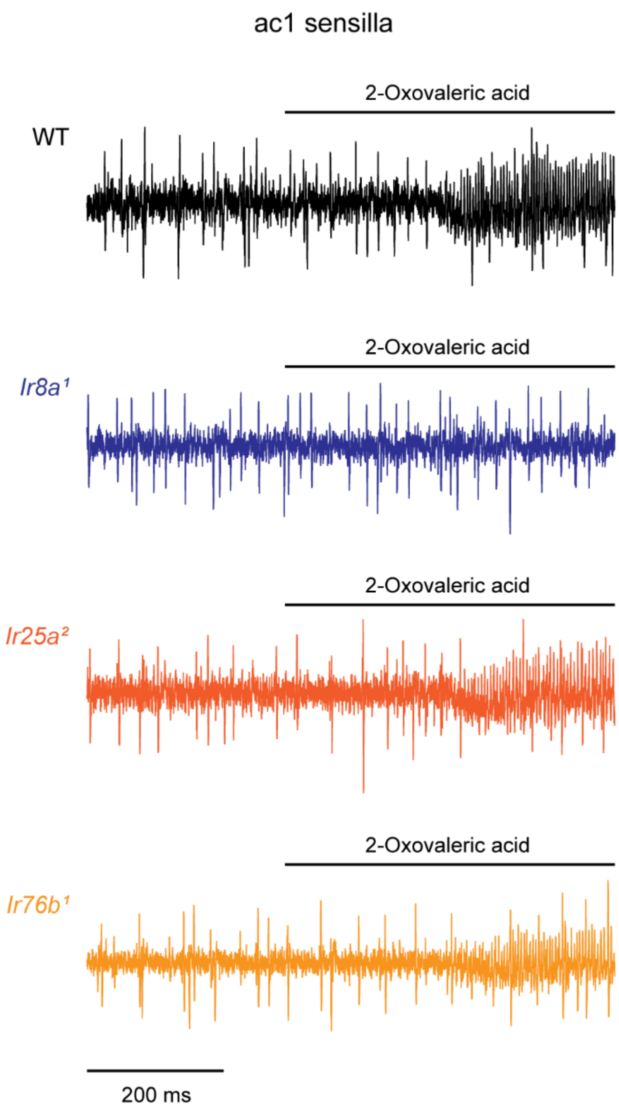

Supplementary Figure S2. Expanded traces for ac1 sensilla. The traces showing responses to 2-oxovaleric acid from Figure 4 are shown in expanded form.

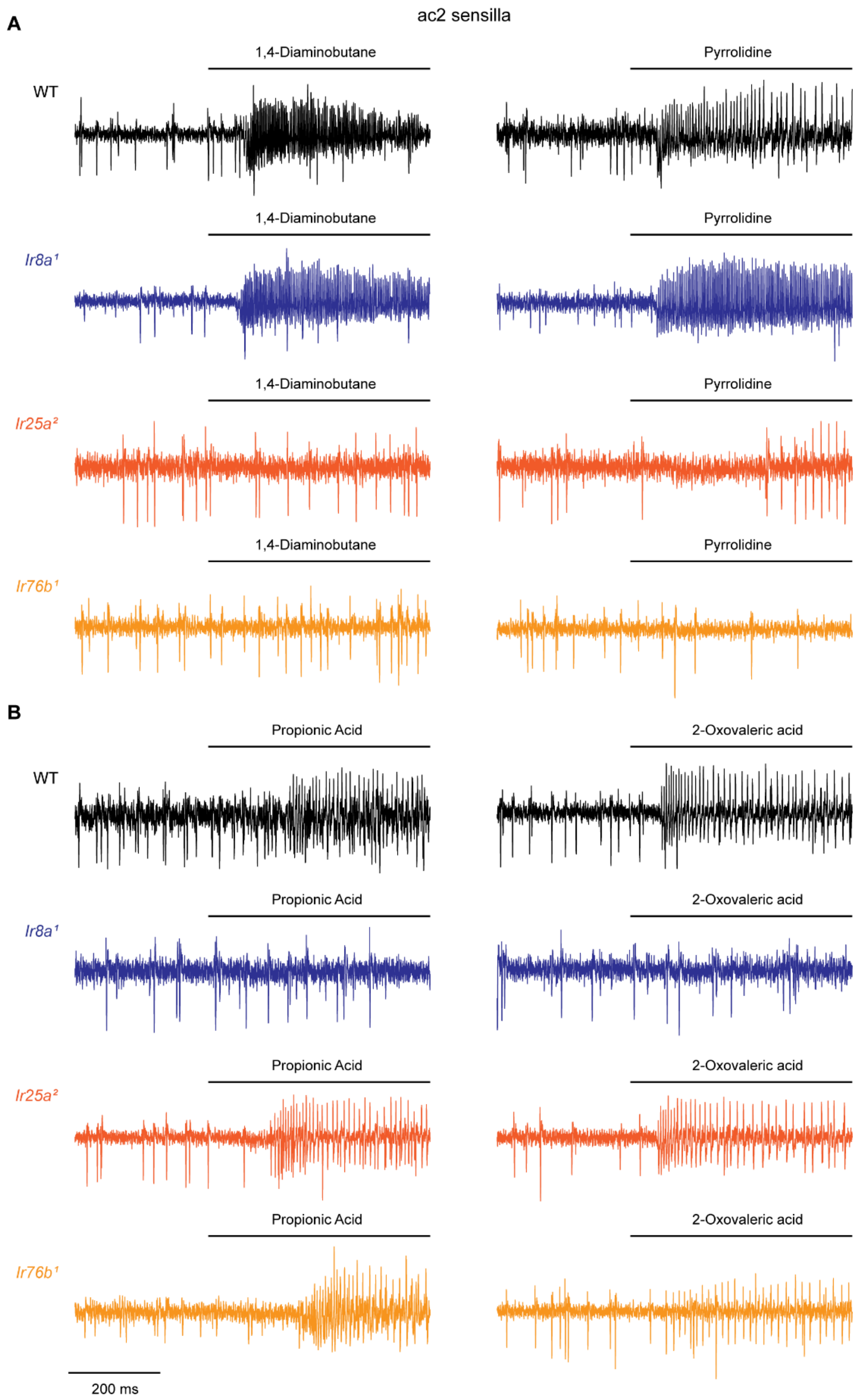

Supplementary Figure S3. Expanded traces for ac2 sensilla. **(A)** The traces showing responses to amines from Figure 1 are shown in expanded form. **(B)** The traces showing responses to propionic acid and 2-oxovaleric acid from Figure 4 are shown in expanded form.

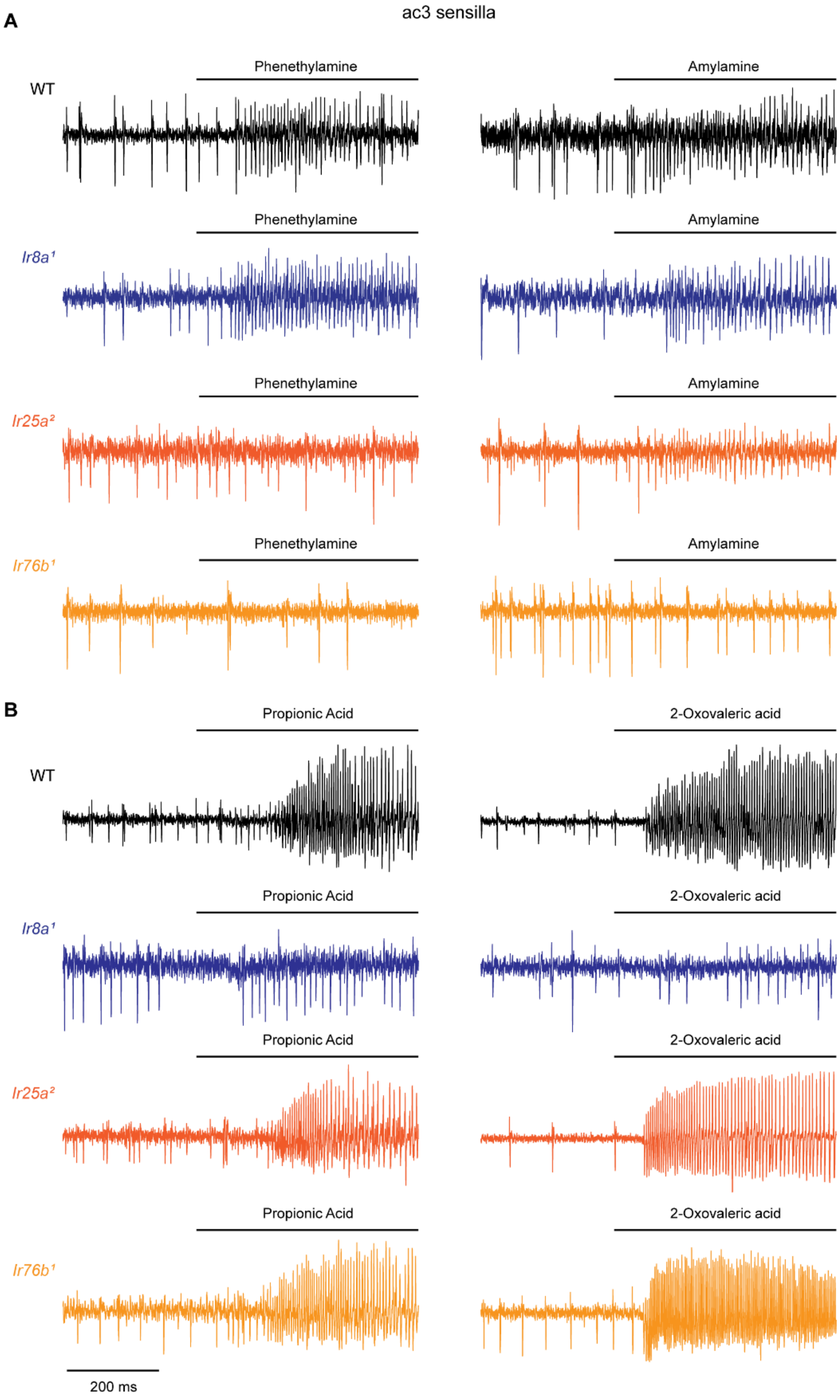

Supplementary Figure S4. Expanded traces for ac3 sensilla. **(A)** The traces showing responses to amines from Figure 3 are shown in expanded form. **(B)** The traces showing responses to propionic acid and 2-oxovaleric acid from Figure 4 are shown in expanded form.

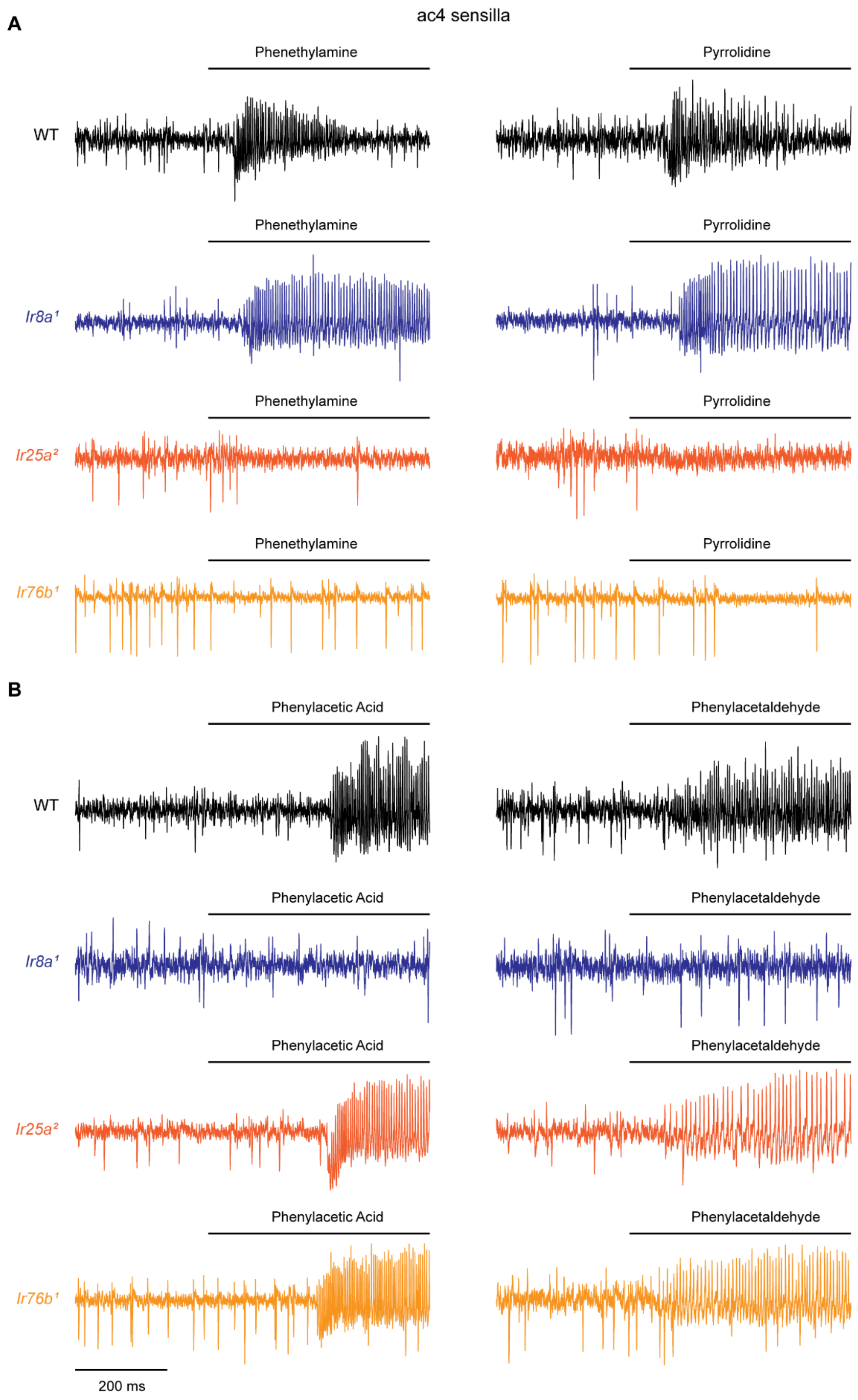

Supplementary Figure S5. Expanded traces for ac4 sensilla. **(A)** The traces showing responses to phenethylamine and pyrrolidine from Figure 1 are shown in expanded form. **(B)** The traces showing responses to phenylacetic acid and phenylacetaldehyde from Figure 4 are shown in expanded form.

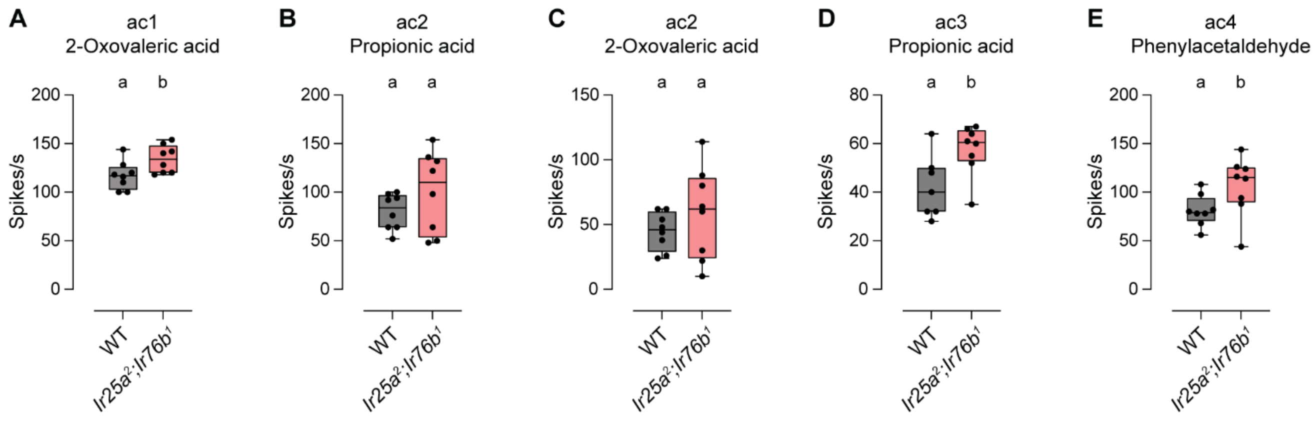

Supplementary Figure S6. Responses to acids in *Ir25a^2;Ir76b^1* flies. **(A)** Responses to 2-oxovaleric acid in ac1 sensilla are slightly increased in *Ir25a^2;Ir76b^1* mutants (n=8 sensilla each). **(B)** Responses to propionic acid in ac2 sensilla are unchanged in *Ir25a^2;Ir76b^1* mutants (n=8 sensilla each). **(C)** Responses to 2-oxovaleric acid in ac2 sensilla are unchanged in *Ir25a^2;Ir76b^1* mutants (n=8 sensilla each). **(D)** Responses to propionic acid in ac3 sensilla are increased in *Ir25a^2;Ir76b^1* sensilla (n=7 WT and 8 *Ir25a^2;Ir76b^1* sensilla). Note, the WT sensilla are a subset of those shown in Figure 4C, and are those recorded in parallel with the *Ir25a^2;Ir76b^1* mutants. **(E)** Responses to phenylacetaldehyde in ac4 sensilla are increased in *Ir25a^2;Ir76b^1* mutants (n=8 sensilla each). In each panel, genotypes that are significantly different are indicated with different letters.
